# Institutional Notes and News

**Published:** 1910-08-27

**Authors:** 


### Institutional Notes and News.

**Sunderland's Children's Hospital.**

At the quarterly meeting of the Sunderland Infirmary, a communication was received from the Secretary of State stating that the message of condolence from the workmen's governors of the Sunderland Infirmary on the death of the late King had been laid before her Majesty Queen Alexandra. The question of the maintenance of the Children's Hospital has had for some time past the consideration of the governors, and the matter was referred to a special sub-committee to consider and report. The sub-committee having held several meetings, unanimously resolved to submit the following recommendation: "That an appeal be made to all sections of employees to contribute one halfpenny per week towards the maintenance of the Children's Hospital." The report having been submitted, was very fully discussed, and afterwards adopted, only one voting against. The governors are unanimous in their opinion that every effort should be made as soon as possible to assist the General Committee in raising sufficient revenue for the maintenance of the hospital, in view of the fact that the building is now completed, and can be opened as soon as the necessary funds are forthcoming. The governors desire their fellow employees to give their hearty support to the proposal now made, recognising as they do that the hospital will be a great blessing to the many sick and suffering children who need treatment such as can only be obtained in a thoroughly equipped and up-to-date hospital.

**Luton Bute Hospital.**

Plans have now been adopted for the much-discussed extension of the Luton Bute Hospital. A new ward for twelve beds will be constructed, thereby raising the accommodation in the main ward block to thirty-six. An open-air verandah is to be built also. The nurses will benefit by a new dining-room, which, we understand, is also to be used as a board room, and new offices, including a dispensary and eight nurses' bedrooms, are now to be added to the administration block. There is to be a new operation theatre, the design of which has been discussed carefully by the medical staff. The cost of these extensions is expected to be £5,000, of which £3,100 is promised or subscribed, so the remainder should be raised by the time the alterations are finished, and so it will be if energy is used to get it. The hospital is built practically on the pavilion system, and the architects, whose names unfortunately have not reached us, are to be congratulated on the foresight by which the walls of the new ward, and of the last before them, have been constructed so as to bear a new story should that be necessary one day. Tenders are not to be advertised for, but invited by the architects and submitted to the building (advisory) committee.

**General Infirmary, Worcester.**

At the meeting of the Executive Committee on Monday, August 14, before proceeding to the business of the day the Chairman moved that a vote of condolence with the family of their late Vice-Chairman, the Rev. Canon Littleton Wheeler, be passed and forwarded to Mrs. Littleton Wheeler. So recently as June, in spite of increasing weakness, he presided at the meeting of the Executive Committee, and the Infirmary, as well as many other charitable institutions, lose a wise and devoted supporter. On the recommendation of the Finance Committee, it was agreed to transfer £1,000 from current account to deposit account, which now stands at £3,000. The reason for this was that the special committee (appointed to discuss the suggestions of the medical staff as to certain much-needed improvements) thought it might be possible to do something if the money were available.

**East Suffolk and Ipswich Hospital.**

At the weekly meeting of the East Suffolk and Ipswich Hospital Board of Management held last week, the Chairman (Mr. F. Messent) read a letter from Dr. J. H. Bartlet enclosing a cheque for £1,000 to endow a bed in memory of his late Majesty King Edward VII. A resolution expressing the Board's grateful thanks was carried unanimously, and the question of procuring a memorial plate was referred to a sub-committee. Dr. Bartlet has ever shown his deep interest in the hospital, of which he is consulting physician as well as President, and this is not his first act of generosity since he accepted the presidential chair. Coming at a time when the capital account has been somewhat depleted, remarks the *East Anglian Times*, the donation of £1,000 is exceptionally welcome, and must be gratifying to governors and general public alike.

**Mansfield Hospital.**

At the annual meeting of delegates in connection with the Mansfield and District Hospital Saturday Fund held last week at Nottingham, the Chairman (Mr. J. H. White) mentioned that a very important decision had been arrived at by the governors at a meeting which was held the previous evening. It was to dispose of the Convalescent Hospital at Mansfield Woodhouse if the governors of the Nottingham Hospital were agreeable, and to devote the proceeds to the extension of the Mansfield Hospital. According to the report in the Nottingham *Daily Express*, Mr. White is stated to have said that the buildings at Mansfield Woodhouse were altogether unsuitable to modern requirements for a hospital, and the whole surroundings had so changed that it was not advisable to continue the present establishment. A number of small cottages had been built near by, and the County Council was proposing to build an elementary school directly opposite. Through Sir Charles Seely, Mr. Hollins had laid the matter before the governors of the Nottingham General Hospital, and they would not offer any objection to the sale if the proceeds were devoted to the purpose named. If the arrangement was carried out they would require more funds, and Mr. White urged those present to use their influence to increase the funds of the hospital to enable expansions to be carried out. The contributions which they were now receiving from the colliers had put the hospital upon a sound financial footing, and they looked to those who received large sums from mineral rents, colliery dividends, and so forth to contribute their fair quota. They had done fairly well in that direction in the past, and in spite of the Budget he hoped that they would continue to contribute liberally.

**A School Boy as Hospital Governor.**

At the monthly meeting of the West Ham Hospital Committee the question of life governors was discussed. The School Children's Hospital Fund had nominated several teachers as life governors, and a member of the committee objected to such wholesale nomination. Mr. A. Govier, in reply, said that the Fund's action was perfectly constitutional, and they were bound to accept the names. A Member: Whoever collects the money has a right to nominate the names. The chairman, continuing, said they must not forget that £250 was handed over for the hospital. The teachers took a great interest in this, and the committee ought not to grudge them these governorships. The names were finally accepted. The Finance Committee reported that its attention had been drawn to the large sums collected by Frank Sturton, a scholar of Central Park Road Council School, East Ham, and recommended that, in recognition of this, the committee should present him with a framed certificate. Mr. Waddell thought the teachers might have nominated this boy a life governor. The secretary read a letter from the headmaster of the school to Mr. Child, recommending that the executive committee of the hospital be asked to recognise the action of this scholar. Out of £6 17s. collected at this school the boy had got £1 8s. 8d.; and out of £5 11s. 5d. subscribed in 1909 he had collected £1 11s. 7d. He never had collected less than 10s. each year. Mr. Waddell moved that the letter be sent to Mr. Child, with the suggestion that the lad be nominated a life governor. It would be an encouragement to other boys in other schools. After some discussion, the motion was agreed to, and Master Churton will probably have the honour of being the youngest hospital governor in the Empire!

**A New Convalescent Home for Women.**

The half-yearly meeting of the Leicester and County Saturday Hospital Society was held in the Out-patients' Department of the Infirmary on Saturday. Sir Edward Wood (president) in the chair, supported by Mr. Sydney Grinson and Mr. J. Gipson Clarke (vice-presidents). Two hundred delegates were present. In submitting the report for the half-year Mr. Woolley, the Secretary, mentioned that 427 men and 189 women had received convalescent treatment, a total of 616, as compared with 491 for the first six months of 1909. The expenditure for the six months was £1,914 2s. 4d., an increase of £221 9s. 5d. for the corresponding period. Eight hundred and fifty-six workpeople employed at thirty-two firms had been added to the list of systematic contributors to the Fund. Sir Edward Wood, in moving the adoption of the report, mentioned that great progress had been made in connection with a convalescent home for women. The sanction of the Court had been obtained to the purchase by the trustees of the late Mr. E. Higgs of land at Woodhouse Eaves, near Leicester, plans had been duly passed, and sanction had now been obtained for the acceptance of a first contract for the foundations. With an additional piece of land purchased by the Society, nine acres had been acquired at a cost of £3,000, and the estimated cost of building and equipping a home for about forty-five female patients was £12,000. Of this sum £8,000 had been sanctioned by the Court from the late Mr. Higgs's estate, and the remainder of the estate (about £2,000), with the accumulated interest, would be applied to the endowment of the home. The balance of the cost of erection and equipment would be supplied from the deposit account of the Society at the bank (£7,200). While they were dealing with a number of consumptive cases, they were only on the fringe of a huge work, and he hoped the Society would not rest content until it had its own sanatorium in the county. The report, having been supported by the vice-presidents, was adopted. The delegates afterwards partook of tea on the invitation of the president and vice-presidents.

